# Association between sleep disorders and alterations in glucose metabolism, insulin, and glucagon in patients with type 2 diabetes

**DOI:** 10.1097/MD.0000000000047409

**Published:** 2026-02-13

**Authors:** Zhixue Song, Huashan Zhao, Xiaoxue Yu, Xinguang Sun

**Affiliations:** a Orthopedic Ward 2, Kailuan General Hospital, Tangshan City, Hebei Province, China; b Urology Ward 1, Kailuan General Hospital, Tangshan City, Hebei Province, China; c Emergency Department, Kailuan General Hospital, Tangshan City, Hebei Province, China; d Urology Ward 2, Kailuan General Hospital, Tangshan City, Hebei Province, China.

**Keywords:** glucagon dysregulation, glucose metabolism, insulin resistance, oral glucose tolerance test, Pittsburgh sleep quality index, sleep disorders, type 2 diabetes mellitus

## Abstract

This retrospective observational study investigated the association between sleep disorders and alterations in glucose metabolism, insulin secretion, and glucagon regulation in patients with type 2 diabetes mellitus (T2DM). A total of 294 patients with T2DM were enrolled between January 2020 and December 2024, including 108 patients with sleep disorders and 186 without. Sleep quality was assessed using the Pittsburgh sleep quality index, with a score >7 indicating poor sleep quality. Clinical and biochemical parameters, including fasting plasma glucose, glycated hemoglobin (HbA1c), serum insulin, and plasma glucagon levels, were analyzed. An oral glucose tolerance test was conducted to evaluate dynamic changes in insulin and glucagon secretion. Compared with patients without sleep disorders, those with poor sleep quality exhibited significantly higher fasting plasma glucose, 2-hour plasma glucose, HbA1c, and homeostasis model assessment of insulin resistance (HOMA-IR) values, alongside lower homeostasis model assessment of β-cell function (HOMA-β) (all *P* <.001). During oral glucose tolerance test, insulin responses were attenuated, and glucagon concentrations remained consistently higher with insufficient suppression at postload time points in the sleep-disorder group. These results indicate that sleep disturbances are closely linked to increased insulin resistance, impaired β-cell function, and dysregulated α-cell activity, poor sleep quality was associated with impaired glucose metabolism.

## 1. Introduction

Sleep disturbances are frequent in type 2 diabetes mellitus (T2DM) and are increasingly recognized as clinically relevant determinants of dysglycemia rather than benign comorbid symptoms. Beyond shared risk factors (age, adiposity, comorbid cardiometabolic disease), convergent evidence indicates that poor sleep quality alters neuroendocrine and autonomic pathways, heightening sympathetic activity, perturbing hypothalamic–pituitary–adrenal signaling, and shifting circadian timing, which together impair insulin sensitivity, blunt glucose‐stimulated insulin secretion, and attenuate glucose-dependent glucagon suppression.^[1,2]^ These disturbances may amplify postprandial hyperglycemia and worsen long-term glycemic control, thereby escalating microvascular and macrovascular risk in T2DM. Contemporary practice statements now emphasize systematic sleep assessment as part of comprehensive diabetes care, reflecting the maturation of this evidence base.^[3,4]^ Epidemiologic and clinic-based studies consistently associate subjective sleep impairment (often quantified by the Pittsburgh sleep quality index, PSQI) with higher HbA1c, elevated fasting and postload glucose, and adverse self-management behaviors in adults with T2DM. While early reports were heterogeneous in design, more recent analyses corroborate a monotonic relation between worse PSQI scores and higher HbA1c, independent of adiposity and medication use.^[5]^ Experimental and short-term human studies demonstrate that sleep loss reduces peripheral insulin sensitivity and modifies metabolic flexibility, providing a biological bridge between subjective sleep measures and glycemic outcomes.^[6]^

Mechanistically, dysregulation of both β- and α-cell function appears central. In T2DM, glucose-stimulated insulin secretion is impaired while α-cell glucose sensing and intra-islet paracrine restraint (by insulin and somatostatin) are blunted, yielding inappropriately elevated glucagon during hyperglycemia. Recent syntheses and cohort data highlight persistent fasting hyperglucagonemia and incomplete glucagon suppression during oral glucose tolerance testing (OGTT) as features that track with deteriorating glycemic trajectories from impaired glucose tolerance to overt T2DM.^[7,8]^ Sleep disruption may exacerbate this phenotype through sympathetic activation and circadian misalignment, further weakening α–β coordination and sustaining hepatic glucose output in the postprandial window. Obstructive sleep apnea (OSA) – a prevalent sleep disorder in T2DM – adds intermittent hypoxia and intrathoracic pressure swings to sleep fragmentation, amplifying insulin resistance and oxidative stress. Although continuous positive airway pressure has variable metabolic effects, recent randomized trials show that the dual GIP/GLP-1 receptor agonist tirzepatide reduces apnea–hypopnea index and hypoxic burden while producing substantial weight loss, underscoring bidirectional therapeutic leverage between sleep and metabolism. These advances support the clinical relevance of identifying and treating sleep pathology within diabetes pathways.^[9,10]^

This study aimed to investigate the association between sleep disorders and alterations in glucose metabolism, insulin secretion, and glucagon regulation in patients with T2DM. The findings aim to inform risk stratification and motivate sleep-targeted interventions as adjuncts to standard metabolic therapy in clinical practice.

## 2. Methods

### 2.1. Study design

This retrospective observational study enrolled patients diagnosed with type 2 diabetes mellitus (T2DM) who received treatment and follow-up at our institution between January 2020 and December 2024. Eligible participants were adults aged 30 to 75 years who met the diagnostic criteria for T2DM as defined by the American Diabetes Association (2023). Only patients with complete clinical and biochemical data – including fasting blood glucose, glycated hemoglobin (HbA1c), serum insulin, and plasma glucagon levels – were included in the analysis. Sleep quality and sleep disorders were evaluated using validated instruments, such as the PSQI or polysomnography (PSG), to ensure accurate assessment of sleep parameters. Patients were excluded if they had type 1 diabetes mellitus, gestational diabetes, or other specific forms of diabetes secondary to endocrine or pancreatic disorders; severe hepatic or renal impairment (defined as alanine aminotransferase or creatinine levels exceeding 3 times the upper normal limit); a history of major psychiatric or neurodegenerative disorders; or current use of sedative-hypnotic medications that could interfere with sleep architecture or glucose metabolism. Additional exclusion criteria included a history of OSA–hypopnea syndrome previously treated with continuous positive airway pressure or surgical interventions, alcohol or substance abuse, and irregular sleep–wake patterns such as shift work. This study was approved by the Ethics Committee of Kailuan General Hospital, Tangshan City, Hebei Province, China (Approval No: KLGH-2020-012). Written informed consent was obtained from all participants.

### 2.2. Assessment of sleep quality using the pittsburgh sleep quality index

Sleep quality was assessed in all participants using the PSQI, a standardized and validated self-report questionnaire developed by Buysse et al (1989).^[11]^ The PSQI evaluates subjective sleep quality and disturbances over the preceding month and has been widely applied in clinical and epidemiological studies involving patients with T2DM. It consists of 19 self-rated items, which are grouped into 7 components: subjective sleep quality, sleep latency, sleep duration, habitual sleep efficiency, sleep disturbances, use of sleep medication, and daytime dysfunction. Each component is scored from 0 to 3, and the sum of the 7 component scores yields a global PSQI score ranging from 0 to 21, with higher scores indicating poorer sleep quality. In this study, a global PSQI score >7 was defined as indicative of a sleep disorder, whereas a score of 7 or less represented normal sleep quality. Because subtype diagnostic data (e.g., insomnia, OSA, circadian rhythm disorders) were not consistently available in the retrospective dataset, sleep-disorder classification could not be further stratified.

### 2.3. Data collection

Clinical and biochemical data were collected from all participants at the time of enrollment. Demographic and baseline characteristics, including age, sex, body mass index (BMI), duration of diabetes, and the presence of comorbidities such as hypertension and dyslipidemia, were obtained through standardized questionnaires and review of medical records. Laboratory parameters, including blood urea nitrogen, serum creatinine, triglycerides, total cholesterol, and low-density lipoprotein cholesterol (LDL-C), were measured after an overnight fast using an automated biochemical analyzer (Hitachi 7600, Tokyo, Japan). Glycated hemoglobin (HbA1c) was determined by high-performance liquid chromatography, and sleep quality was assessed using the PSQI.

For the evaluation of glucose metabolism, venous blood samples were collected after at least 8 hours of fasting and again at 2 hours following a 75-g oral glucose load to measure fasting plasma glucose (FPG) and 2-hour plasma glucose (2-h-PG) levels. Insulin resistance and β-cell function were calculated using the homeostasis model assessment indices: HOMA-IR = (FPG × Fasting insulin)/ 22.5; HOMA-β = 20 × Fasting insulin/ (FPG – 3.5).

To further assess dynamic changes in glucose-regulatory hormones, a standard OGTT was conducted. Blood samples were collected at 0, 30, 60, 120, and 180 minutes after glucose ingestion to measure plasma insulin and glucagon concentrations. Insulin levels were determined by a chemiluminescent immunoassay, while plasma glucagon concentrations were quantified using a validated enzyme-linked immunosorbent assay (ELISA; Mercodia, Sweden). All samples were processed within 30 minutes of collection and stored at − 80°C until analysis.

### 2.4. Statistical analysis

All statistical analyses were performed using IBM SPSS Statistics for Windows, version 26.0 (IBM Corp., Armonk). Prior to analysis, data were examined for normality using the Kolmogorov–Smirnov test. Continuous variables with a normal distribution were expressed as mean ± standard deviation, while those with a skewed distribution were presented as median (interquartile range, IQR). Categorical variables were summarized as frequency and percentage (%). Comparisons between the sleep-disorder group and the non–sleep-disorder group were conducted using the independent-samples t-test for normally distributed continuous variables and the Mann–Whitney *U* test for non-normally distributed data. Chi-square (χ^2^) tests were used to compare categorical variables such as sex, hypertension, and dyslipidemia. All statistical tests were 2-tailed, and a *P*-value <.05 was considered statistically significant.

## 3. Results

### 3.1. Baseline characteristics of patients with type 2 diabetes according to sleep status

A total of 294 patients with T2DM were included in the study, comprising 186 patients in the non–sleep-disorder group and 108 patients in the sleep-disorder group. As shown in Table 1, there were no significant differences between the 2 groups in terms of age, sex distribution, BMI, duration of diabetes, prevalence of hypertension or dyslipidemia, or baseline renal and lipid parameters including blood urea nitrogen, serum creatinine, triglycerides, total cholesterol, and LDL-C (*P* >.05 for all). However, patients with sleep disorders exhibited significantly higher glycated hemoglobin (HbA1c) levels compared with those without sleep disorders (8.14 ± 1.27% vs 7.62 ± 1.10%, *P* <.001), indicating poorer long-term glycemic control. In addition, the PSQI score was markedly elevated in the sleep-disorder group (10.6 ± 3.1 vs 5.4 ± 1.7, *P* <.001), confirming the presence of significant sleep quality impairment (Table 1).

**Table 1:** Baseline characteristics of patients with type 2 diabetes mellitus according to sleep status.

Variable	Non-sleep disorder group (n = 186)	Sleep disorder group (n = 108)	Statistics (*t*/χ2)	*P*-value
Age (year)	55.7 ± 8.9	56.8 ± 9.4	1.001	.318
Male, n (%)	108 (58.1%)	64 (59.3%)	0.040	.841
BMI (kg/m^2^)	26.9 ± 3.3	27.4 ± 3.5	1.225	.222
Duration of diabetes (year)	7.9 ± 3.8	8.6 ± 4.2	1.464	.144
Hypertension, n (%)	104 (55.9%)	68 (63.0%)	1.398	.237
Dyslipidemia, n (%)	110 (59.1%)	72 (66.7%)	1.641	.200
BUN (mmol/L)	6.1 ± 1.4	6.3 ± 1.5	1.150	.251
Serum creatinine (μmol/L)	77.8 ± 17.5	79.6 ± 18.2	0.838	.403
Triglycerides (mmol/L)	1.88 ± 0.68	1.94 ± 0.70	0.722	.471
Total cholesterol (mmol/L)	5.21 ± 0.86	5.28 ± 0.89	0.664	.507
LDL-C (mmol/L)	2.98 ± 0.69	3.06 ± 0.71	0.948	.344
HbA1c (%)	7.62 ± 1.10	8.14 ± 1.27	3.689	<.001
PSQI score	5.4 ± 1.7	10.6 ± 3.1	18.58	<.001

BMI = body mass index, BUN = blood urea nitrogen, HbA1c = glycated hemoglobin, LDL-C = low-density lipoprotein cholesterol, PSQI = Pittsburgh sleep quality index.

### 3.2. Glucose metabolism parameters in patients with type 2 diabetes according to sleep status

As shown in Table 2, significant differences were observed in multiple indicators of glucose metabolism between patients with and without sleep disorders. The sleep-disorder group exhibited markedly higher FPG (8.63 ± 1.57 vs 7.84 ± 1.45 mmol/L, *P* <.001) and 2-hour postload plasma glucose levels (13.78 ± 2.61 vs 12.36 ± 2.42 mmol/L, *P* <.001), suggesting poorer glycemic control and reduced glucose tolerance. Similarly, HbA1c levels were significantly elevated in the sleep-disorder group (8.18 ± 1.25% vs 7.61 ± 1.09%, *P* <.001), indicating chronic hyperglycemia and suboptimal long-term glucose regulation. In addition, the homeostasis model assessment of insulin resistance (HOMA-IR) was significantly higher among patients with sleep disorders (4.29 ± 1.21 vs 3.68 ± 1.03, *P* <.001), reflecting an increased degree of insulin resistance. Conversely, the homeostasis model assessment of β-cell function (HOMA-β) was lower in this group (54.2 ± 15.3 vs 62.3 ± 16.1, *P* <.001), suggesting impaired pancreatic β-cell secretory capacity.

**Table 2:** Comparison of glucose metabolism parameters between groups.

Variable	Non–sleep disorder group (n = 186)	Sleep disorder group (n = 108)	*t*-value	*P*-value
Fasting plasma glucose (mmol/L)	7.84 ± 1.45	8.63 ± 1.57	4.368	<.001
2-h plasma glucose (mmol/L)	12.36 ± 2.42	13.78 ± 2.61	4.711	<.001
HbA1c (%)	7.61 ± 1.09	8.18 ± 1.25	4.093	<.001
HOMA-IR	3.68 ± 1.03	4.29 ± 1.21	4.586	<.001
HOMA-β	62.3 ± 16.1	54.2 ± 15.3	4.235	<.001

HOMA-IR = homeostasis model assessment of insulin resistance, HOMA-β = homeostasis model assessment of β-cell function.

### 3.3. Plasma insulin response during oral glucose tolerance test

The dynamic changes in plasma insulin concentrations during the OGTT are presented in Table 3. At baseline (0 minutes), fasting insulin levels were significantly higher in the sleep-disorder group compared with those without sleep disorders (15.3 ± 6.1 vs 13.7 ± 5.6 μIU/mL, *P* = .023), indicating a tendency toward insulin resistance. Following glucose ingestion, both groups demonstrated a rapid increase in insulin levels, reaching a peak at 60 minutes. However, insulin concentrations at 30, 60, 120, and 180 minutes were consistently lower in the sleep-disorder group compared with the non–sleep-disorder group (*P* <.01 for all time points). The attenuated postload insulin secretion and delayed decline suggest that patients with sleep disorders exhibit impaired β-cell responsiveness to glucose stimulation.

**Table 3:** Plasma insulin concentrations (μIU/mL) during OGTT.

Time point	Non–sleep disorder group (n = 186)	Sleep disorder group (n = 108)	*t*-value	*P*-value
0 min	13.7 ± 5.6	15.3 ± 6.1	2.285	.023
30 min	54.8 ± 17.9	48.6 ± 18.3	2.840	.005
60 min	70.1 ± 20.8	63.4 ± 21.6	2.625	.009
120 min	59.2 ± 18.7	52.7 ± 19.5	2.828	.005
180 min	39.5 ± 14.1	34.7 ± 13.8	2.836	.005

OGTT = oral glucose tolerance test.

### 3.4. Plasma glucagon concentrations during oral glucose tolerance test

As shown in Table 4, plasma glucagon concentrations were consistently higher in patients with sleep disorders compared with those without sleep disorders at all time points during the OGTT. At baseline (0 minutes), fasting glucagon levels were significantly elevated in the sleep disorder group (100.5 ± 30.6 vs 86.3 ± 21.8 ng/L, *P* <.001), indicating increased basal α-cell activity. After glucose ingestion, glucagon levels initially rose and then gradually declined in both groups; however, the extent of suppression was notably reduced in the sleep disorder group. At 30 and 60 minutes, glucagon concentrations remained markedly higher among patients with sleep disorders (*P* <.01), and this difference persisted through 120 and 180 minutes (*P* <.05). The impaired glucagon suppression observed throughout the test suggests dysregulated α-cell function and diminished responsiveness to glucose and insulin signals in patients with sleep disturbances.

**Table 4:** Plasma glucagon concentrations (ng/L) during OGTT.

Time point	Non–sleep disorder group (n = 186)	Sleep disorder group (n = 108)	*t*-value	*P*-value
0 min	86.3 ± 21.8	100.5 ± 30.6	4.625	<.001
30 min	125.7 ± 29.4	139.8 ± 26.7	4.098	<.001
60 min	146.5 ± 31.8	156.9 ± 32.4	2.685	.008
120 min	156.7 ± 28.5	168.4 ± 37.1	2.426	.016
180 min	111.9 ± 35.2	127.3 ± 39.5	3.456	.001

OGTT = oral glucose tolerance test.

## 4. Discussion

This study demonstrates that, among adults with T2DM, poor sleep quality – indexed by higher PSQI scores – is associated with adverse alterations across the glucose–insulin–glucagon axis. At baseline, groups were comparable in age, sex distribution, BMI, diabetes duration, and standard renal and lipid indices, minimizing confounding by these variables. Nevertheless, the sleep-disorder group exhibited higher HbA1c and consistently worse glycemic indices (fasting glucose and 2-h postload glucose), together with higher HOMA-IR and lower HOMA-β. During OGTT, the sleep-disorder group showed a pattern of modestly higher fasting insulin but blunted postload insulin excursions and persistently higher plasma glucagon with attenuated suppression from 30 to 180 minutes. These findings indicate that sleep disturbance in T2DM is accompanied by greater insulin resistance, impaired β-cell responsiveness, and dysregulated α-cell activity, which together favor hyperglycemia.

Our study is among the few to simultaneously examine dynamic insulin and glucagon profiles during OGTT in relation to sleep quality in T2DM. Poor sleep can increase sympathetic activity and activate the hypothalamic–pituitary–adrenal axis, thereby raising hepatic glucose output and reducing insulin sensitivity. Chronic sleep disruption may also alter metabolic hormones. Our findings are consistent with these mechanisms: higher HOMA-IR and elevated fasting insulin in the sleep-disorder group suggest compensatory insulin secretion, while reduced early and peak insulin responses during OGTT indicate impaired glucose-stimulated β-cell function. At the same time, persistently higher glucagon levels and insufficient postload suppression reflect α-cell dysregulation and weakened intra-islet paracrine control. Given that α-cell glucose sensing is already impaired in T2DM, sleep disturbance may further limit glucagon suppression, contributing to increased hepatic glucose production after glucose loading.^[12,13]^

The OGTT-based hormone dynamics strengthen this interpretation. In the non–sleep-disorder group, insulin rose briskly and then declined, while glucagon showed the expected suppression. In contrast, participants with sleep disturbance displayed attenuated insulin peaks and persistently elevated glucagon throughout recovery. This reciprocal disturbance implies weakened α–β coordination: insufficient insulin-mediated paracrine inhibition of α-cells and reduced somatostatin restraint would favor persistent glucagonemia despite hyperglycemia. Such a pattern predicts a larger glucose area under the curve, delayed return toward baseline, and higher HbA1c – features observed in the current cohort. These findings also carry implications for heterogeneity within “sleep disorders.” OSA, insomnia symptoms, and short/irregular sleep likely contribute through both shared and distinct mechanisms.^[14,15]^ Recurrent intermittent hypoxia, intrathoracic pressure swings, and sleep fragmentation in OSA amplify sympathetic drive and oxidative stress, promoting hepatic glucose output and insulin resistance; insomnia and curtailed sleep may impair slow-wave sleep–dependent β-cell function and reduce insulin sensitivity independent of hypoxia. Although OSA was not separately adjudicated here, the directionality of our results is consistent with these pathophysiological models and suggests that multiple sleep phenotypes could converge on a common metabolic signature characterized by insulin resistance, impaired insulin secretory dynamics, and insufficient glucagon suppression.

Recent clinical and population studies reinforce the link between sleep quality and dysglycemia. A 2024 Diabetes Care editorial synthesized contemporary evidence and emphasized sleep’s central role across the T2DM spectrum, noting that poorer sleep quality is consistently associated with worse glycemic control and higher HbA1c, and calling for routine sleep assessment in diabetes care. More recently, cohort data from 2025 reported inverse associations between PSQI-derived sleep quality and glycemic indicators (HbA1c, HOMA-IR) in adults with diabetes, dovetailing with our observation that worse PSQI is accompanied by higher insulin resistance and lower HOMA-β.^[16]^ Meta-analytic and narrative syntheses published in 2025 further describe a robust association between insomnia-related symptoms and adverse glycemic control, extending findings beyond apnea to broader sleep disturbance phenotypes; these reports align with our results showing higher fasting and postload glucose in those with poorer sleep.^[17]^

With respect to α-cell function, current work highlights the importance of glucagon dysregulation across the dysglycemia continuum. A 2024 synthesis concluded that inadequate glucagon suppression during OGTT is evident even in prediabetes, implicating α-cell dysfunction as a contributor to both impaired fasting glucose and impaired glucose tolerance – an observation that contextualizes the persistent hyperglucagonemia we observed in T2DM with sleep disturbance.^[18]^ On the mechanistic front, a 2023 review of α-cell electrophysiology summarizes how intrinsic ion-channel properties and paracrine inputs govern glucose-dependent glucagon secretion; failure of these brakes, as may occur with chronic sympathetic activation and β-cell insufficiency, produces the pattern of incomplete suppression documented here.^[19]^ Finally, modeling and conceptual analyses in 2025 emphasize that varying degrees of α-cell dysregulation materially shape trajectories of fasting glucose, 2-h glucose, and HbA1c during T2DM progression, consistent with the stronger postload hyperglycemia in our sleep-disorder group.^[20]^ The intersection of sleep-disordered breathing and metabolic control is also evolving. While our study did not phenotype OSA, recent clinical advances indicate that therapies which reduce weight and improve OSA severity – such as tirzepatide – can meaningfully modify sleep pathophysiology; these developments underscore bidirectional links between sleep and metabolism and the potential for dual-benefit interventions in appropriate patients. Taken together, contemporary literature supports our principal findings: poor sleep quality in T2DM aligns with higher HbA1c, greater insulin resistance, impaired β-cell response, and insufficient glucagon suppression during glucose challenge.

The co-occurrence of higher HOMA-IR and blunted insulin secretion suggests both peripheral and pancreatic components. Sympathetic activation, circadian misalignment, and intermittent hypoxia (in subsets with OSA) can impair insulin signaling in skeletal muscle and liver, while sleep fragmentation may reduce slow-wave sleep, a stage implicated in glymphatic function and neuroendocrine regulation of β-cell activity. Reduced intra-islet insulin at critical early time points would diminish paracrine inhibition of α-cells, plausibly explaining the sustained glucagon levels seen after glucose ingestion. The clinical expression is higher fasting and 2-h glucose and elevated HbA1c, even when traditional cardiometabolic covariates are comparable. From a therapeutic perspective, these data argue for routine identification and management of sleep disturbance within diabetes care pathways. Behavioral and device-based strategies (sleep hygiene, cognitive behavioral therapy for insomnia, screening and treatment for OSA) may provide metabolic benefit by lowering sympathetic tone, normalizing circadian signaling, and improving insulin sensitivity. Pharmacologic agents that reduce body weight and ameliorate OSA severity may further assist selected patients, although evidence specific to α-cell regulation remains limited and deserves targeted study.

The present findings support integrating sleep assessment into routine T2DM management. Elevated PSQI scores identify patients at risk for higher HbA1c, greater insulin resistance, and impaired postload hormone dynamics. Systematic screening for sleep disturbances, including OSA, should be considered, followed by tailored interventions (behavioral sleep therapy, weight loss, OSA treatment). In clinical counseling, attention to sleep timing and regularity may complement diet and activity advice. For patients with persistent postprandial hyperglycemia despite standard therapy, evaluation of sleep health may uncover modifiable factors contributing to inadequate glucagon suppression and suboptimal insulin responses, enabling a more comprehensive, mechanism-informed treatment plan. This study has several strengths. First, we examined the glucose–insulin–glucagon triad using repeated sampling during a standardized OGTT, allowing simultaneous assessment of β-cell responsiveness and α-cell suppression – features rarely captured together in clinical cohorts. Second, groups were balanced across key demographic and metabolic covariates, limiting confounding by obesity, renal function, and lipid status. Third, sleep quality was quantified using a validated instrument (PSQI), enabling reproducibility and comparability with other cohorts.

This study has several limitations that should be acknowledged. First, as a retrospective observational analysis from a single center, causal relationships cannot be inferred, and the findings may not be fully generalizable to other populations or clinical settings. Second, sleep quality was assessed using the PSQI alone, and detailed diagnoses of sleep disorder subtypes – such as insomnia, OSA, or circadian rhythm disturbances – were not available; therefore, potential heterogeneity across sleep phenotypes could not be evaluated. Third, objective sleep measures, including polysomnography or actigraphy, were not included, which may lead to misclassification of sleep status. Fourth, although biochemical testing was conducted using consistent instruments and standardized procedures, residual batch effects or unmeasured variations across the 5-year data collection period cannot be entirely excluded. Fifth, potential confounding factors – such as depressive symptoms, autonomic dysfunction, medication use affecting sleep or glucose metabolism, and lifestyle variables – were not fully assessed. Finally, dynamic hormone measurements were limited to insulin and glucagon during OGTT; additional biomarkers (e.g., incretins, cortisol, sympathetic activity markers) were not available, restricting deeper mechanistic interpretation.

## 5. Conclusions

In patients with type 2 diabetes mellitus, poor sleep quality was significantly associated with impaired glucose metabolism, higher insulin resistance, and dysregulated glucagon secretion. Compared with individuals without sleep disorders, those with sleep disturbances exhibited higher fasting and postprandial glucose levels, elevated HbA1c, and insufficient glucagon suppression during OGTT. These findings suggest that sleep disturbances may exacerbate metabolic dysfunction and should be a key focus in diabetes management.

## Author contributions

**Conceptualization:** Zhixue Song, Huashan Zhao, Xiaoxue Yu, Xinguang Sun.

**Data curation:** Zhixue Song, Huashan Zhao, Xiaoxue Yu, Xinguang Sun.

**Formal analysis:** Zhixue Song, Huashan Zhao, Xiaoxue Yu, Xinguang Sun.

**Funding acquisition:** Zhixue Song, Xinguang Sun.

**Investigation:** Zhixue Song, Xinguang Sun.

**Writing – original draft:** Huashan Zhao, Xiaoxue Yu, Xinguang Sun.

**Writing – review & editing:** Huashan Zhao, Xiaoxue Yu, Xinguang Sun.
